# Use of Antimicrobials in a French Veterinary Teaching Hospital: A Retrospective Study

**DOI:** 10.3390/antibiotics10111369

**Published:** 2021-11-09

**Authors:** Caroline Prouillac

**Affiliations:** VetAgro Sup Campus Vétérinaire de Lyon, Université de Lyon, 69280 Marcy l’Etoile, France; caroline.prouillac@vetagro-sup.fr

**Keywords:** antibiotics, antimicrobial resistance, prescription, monitoring

## Abstract

Antibiotic resistance has become a major concern for not only human health, but also for animal health. To preserve the efficacy of antibiotics, it has become essential to establish measures to regulate the prescription of antibiotics to ensure their prudent use. In France, these measures have been translated into regulations for animal health since 2015, with the publication of three important regulatory texts. The results obtained on a national scale in terms of reducing the use of antibiotics have been satisfactory. The aim of our study was to evaluate the differences related to the prescription of antimicrobials at the veterinary teaching hospital of the Veterinary School of Lyon (CHUV) before and after the implementation of French regulations. Prescriptions and consumption of antimicrobials were examined, along with bacteriological analyses, for the period of 2014–2020, for companion animals and horses. The most frequently prescribed compounds were broad-spectrum antimicrobials, including penicillins with β-lactamase inhibitors, as well as first-generation cephalosporins tetracyclines and sulfamides. The prescription and consumption of critically important antibiotics (CIA) strongly decreased during the study period, with an increase of bacteriological analyses. This study shows the interest of having computerized tools to monitor the use of antimicrobials to implement corrective measures if needed.

## 1. Introduction

Antimicrobial use (AMU) and antimicrobial resistance (AMR) in humans and animals are increasingly becoming health concerns, and public awareness of AMR has grown. Addressing AMR can only be achieved through a One Health approach [1,2]. Use and overuse of antimicrobials in multiple sectors (human, animal, agriculture) is at the origin of the appearance of antibiotic resistance, reducing the effectiveness of antimicrobial therapy in animal and human health. All sectors are interconnected and the transmission of resistance carriers and/or resistant bacteria between each sector has been demonstrated. The One Health approach includes consideration of the environment as well as human and animal health. This concept tends to improve antimicrobial stewardship in humans and animals as is recommended by the Tripartite Alliance formed in 2010 between the World Organisation for Animal Health (OIE), the Food and Agriculture Organization of the United Nations (FAO) and the World Health Organization (WHO) [3,4,5].

Veterinarians and physicians have become very involved in the management of risk related to inappropriate antibiotic use or administration of antimicrobials at inappropriate dosages and administration rate. The use of antibiotics in animal husbandry is often considered responsible for the transmission of resistant germs to humans, especially through foodstuffs [6,7,8]. However, some studies reveal the presence of resistant bacteria in pets [9,10], which are likely to be transmitted to humans [11,12,13,14] or may be of human origin, as in the case of methicillin-resistant *Staphylococcus aureus* (MRSA) [15,16,17].

**Figure 1:**
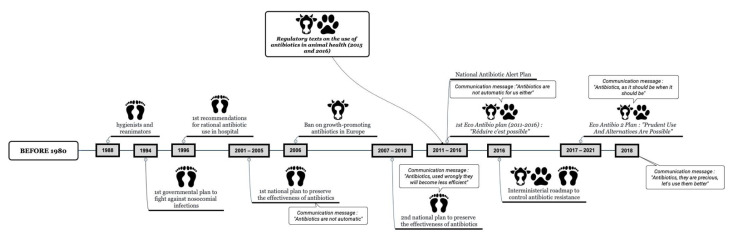
Chronology of the different action plans in human and animal health with communication announcement (footprints: human health; paws and bovine faces: animal health).

In human medicine, a variety of strategies for controlling or changing patterns of antimicrobial use have been attempted to control resistance while taking in account that once bacteria become resistant, redeveloping susceptibility to antimicrobial therapy is not guaranteed and is a lengthy process, even if selection pressure is decreased [18]. In France, the first organized actions to combat bacterial resistance in human medicine began in 1988 with the fight against nosocomial infections due to multi-resistant bacteria like MRSA (Figure 1). The resulting government plan was created in 1994 [19]. The first recommendations about the rational use of antibiotics were published in 1996 by hospital physicians, and a government plan to preserve antibiotic efficacy began in 2001 [20,21]. In parallel, local antibiotic committees have been appointed in each hospital, along with antibiotic authorities who are in charge of giving advice and controlling antibiotic therapy [22].

In 2016, an interministerial roadmap was established to address AMR [23]. This roadmap marked the beginning of the One Health approach to the management of AMR. It was built around five axes: increasing public awareness, improving the use of antibiotics, increasing support for research and innovation, and strengthening surveillance and France’s commitment to the international fight against antibiotic resistance.

The animal health sector took up the subject later with the Ecoantibio Plans (EcoAntibio 2007–2012 and Ecoantibio 2017–2021), which involve public policy set up by the Ministry of Agriculture, Agro-Food, and Forestry to reduce the risks of antibiotic resistance used in veterinary medicine and to safeguard the efficacy of antibiotics used in human medicine [24,25]. This action plan has allowed for the publication of regulatory texts to reduce indiscriminate use of antibiotics and to improve AMU practices [26]. Veterinary professional organizations have also published guides for the rational use of antibiotics, including preventive measures and effective infection control [27].

Based on the regulations, veterinarians can write prescriptions or dispense antibiotic drugs under certain conditions, of which the main ones are: (1) prescribing after clinical examination (2) preventive use of antimicrobials should be avoided or restricted to certain conditions, and (3) avoiding off-label use and, if it is not possible, adapting the dosage regimen based on scientific considerations. In 2005, the World Health Organization (WHO) established a classification of critically important antimicrobials for human medicine due to non-human use. This list is regularly revised until 2018 [28]. The World Organisation for Animal Health (OIE) adopted a list of antimicrobial agents of veterinary importance in May 2007, which was regularly updated until 2018 by the World Assembly of OIE Delegates [29]. Based on these proposition, the French government has established a regulatory list of critically important antibiotics (CIAs) in human and veterinary medicine [30,31]. This publication has led to two major obligations for veterinarians: (1) use of human CIAs is forbidden in veterinary medicine with the exception of some fluoroquinolones administered by ocular routes and (2) the prescription of veterinary CIAs must be justified by the results of susceptibility tests, regardless of the indication and the route of administration. Although veterinary school might be an example of a rational use of antibiotics, there has been no research or surveillance on the current use of antibiotics in French teaching veterinary hospitals and no antibiotic managers as in human hospitals, although some studies on the topic have been published in other countries [32,33,34].

These regulations, which include financial and penal sanctions (especially when no susceptibility test results are available to justify CIA prescription), are at the origin of a modification of practices and teaching. The aim of this study was to evaluate the impact of antimicrobial use legislation on antimicrobial prescription, delivery, and animal level of exposure to antimicrobials (ALEA) at the teaching veterinary school of Lyon (CHUV) in France from 2014 to 2020. This study focused only on companion animals and horses.

## 2. Results

### 2.1. Overall Antimicrobials Prescriptions

From 2014 to 2020, 14,370 antimicrobial prescriptions were written for 9979 animals (dogs, cats, horses, and uncommon pets). There was a small decrease in hospital activity in 2020 due to the COVID-19 crisis. The number of prescriptions has gradually increased since 2014, particularly in the equine sector. In 2012, the software used was updated to allow clinicians to write prescriptions, and the use of this add-on has gradually become more widespread in this clinical sector. Farm animals were excluded from this analysis because this clinical sector did not use the software to write prescriptions.

In the years of the study period, the average antimicrobial prescriptions represented 24.0 ± 3.3% of total prescriptions (Table 1). There was a significant decrease in antibiotic prescriptions between 2014 and 2015. In the following years, the level of prescription frequency was relatively constant. Conversely, the proportion of animals for which an antibiotic was prescribed significantly increased between 2014 and 2015 and stabilized in 2015, reaching a mean of 17.6 ± 2.7%.

Most antimicrobial prescriptions contain only one antibiotic (83.9 ± 2.9%), but this proportion significantly decreased between 2014 and 2015. In addition, the frequency of prescriptions containing several antibiotics significantly increased from 2015, despite a stable frequency of prescriptions containing only one antibiotic. Generally, prescriptions containing several antibiotics include different routes of administration.

Finally, the number of prescriptions containing a CIA decreased significantly between 2014 and 2016 to a level that was relatively stable through 2020.

Dogs were the most-prescribed-to species, with a higher average frequency of antibiotic prescription than cats, but lower than horses (Table 2). Concerning uncommon pets, the average frequency of antibiotic prescriptions was close to that of canine species, but globally, it remains much lower than in 2014. Overall, regardless of the species, the frequency of antibiotic prescriptions significantly decreased since 2015 and stabilized in subsequent years.

**Figure 2:**
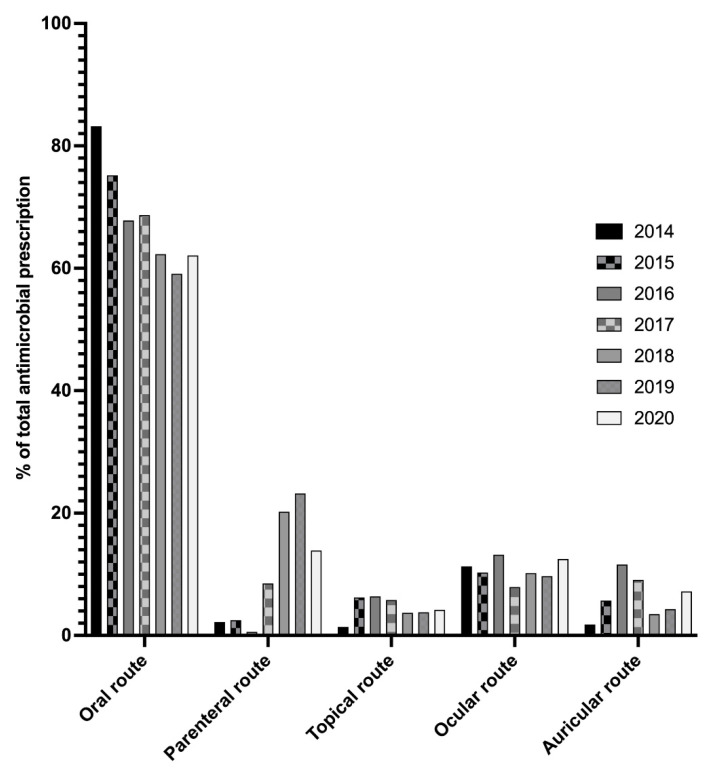
Distribution of antimicrobial prescription by route of administration of antibiotic presentations during 2014 through 2020.

The oral route was the main route of administration of prescribed antibiotics regardless of the year and species (Figure 2). A trend towards a decrease in the oral route and an increase in the proportion of the parenteral route was observed over the study period. External and local routes remained in the minority.

**Figure 3:**
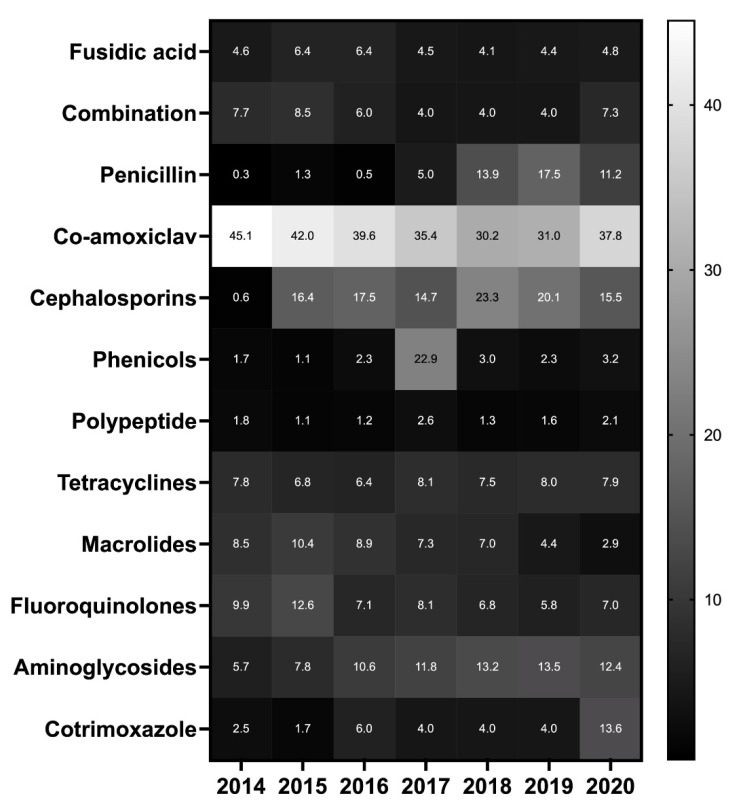
Distribution of class of antibiotic—all species combined—from 2014 to 2020, regardless of route of administration and pathology.

Regarding the class of antibiotics, the most frequently prescribed was the combination of amoxicillin and clavulanic acid, followed by cephalosporins, until 2020. In 2020, there was an increase of cotrimoxazole prescriptions (Figure 3). Tetracyclines, macrolides and fluoroquinolones were the second most common.

### 2.2. Data on Critically Important Antibiotics

**Figure 4:**
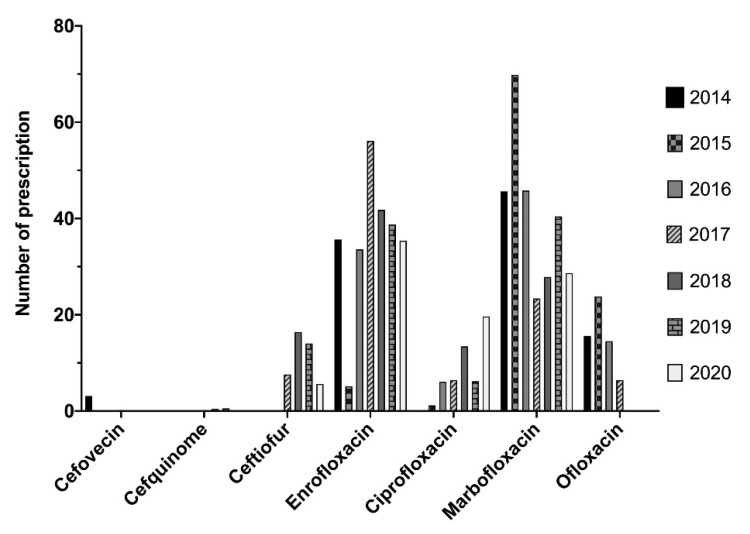
Distribution of CIAs during 2014 through 2020 regardless of species and route of administration.

The critically important antibiotics authorized in veterinary medicine are cephalosporins (cefovecin, cefquinome, ceftiofur) and the latest-generation fluoroquinolones (Figure 4). Cefovecin is strictly used in carnivores, while cefquinome and ceftiofur are prescribed only for horses. Concerning these drugs, we observed a clear decrease of their prescriptions in the respective species. The apparent increase observed with ceftiofur and cefquinome was correlated with the beginning of our use of software to prescribe medicine for horses.

Enrofloxacin and marbofloxacin were mostly used in carnivores, in accordance with market authorization. Some prescriptions of enrofloxacin have been made for horses. Globally, the prescriptions of these two molecules have clearly decreased during the analysis period. In 2015, there was a great decrease in enrofloxacin prescriptions, which was compensated by prescriptions of marbofloxacin.

Concerning ciprofloxacin and ofloxacin, these molecules are used in human medicine and authorized for animals only by ocular route. Prescriptions of these two molecules were essentially by ocular route in horses for ciprofloxacin and by auricular route for ofloxacin in dogs, especially in the context of otitis. Only ciprofloxacin prescriptions tended to decrease during the analyzed period.

**Figure 5:**
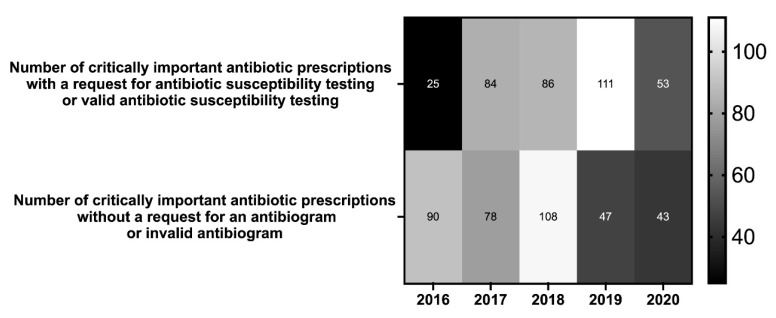
Distribution of CIAs from 2014 to 2020 regardless of species and route of administration.

Prescription of CIAs must be justified, particularly by susceptibility test results. For each CIA prescription, we looked for the presence or absence of antibiogram results. The results are reported in Figure 5. The data show that requests to perform an antibiotic susceptibility test gradually increased from 2016 through 2019, with a net decrease in 2020, while the number of prescriptions did not decrease. Conversely, prescriptions that are not associated with this request or for which the antibiogram is no longer valid have decreased (with a peak in 2018).

Veterinarians were asked to fill a questionnaire for each prescription of CIAs. The questionnaires only checked several proposals related to the reason for the prescription and the availability of sensibility test results. The results showed a gradual increase in the number of completed questionnaires from 2016 to 2019, with a decrease in 2020, but the number of prescriptions did not decrease overall (Table 3). In about 60% of the cases, the veterinarians justified the prescription by the absence of other suitable and effective antibiotics, independently of the presence of bacteriology results. The use of CIAs in a probabilistic context increased to 38.4% in 2020. The results of bacteriological identification associated with CIA prescription were searched (Table 4). The data suggest a high prevalence of *Pseudomonas aeruginosa* in ear-swab samples of dogs. Secondarily, *Staphylococcus* spp. were isolated in otitis in the majority of cases. The most frequently prescribed antibiotics against these bacteria are fluoroquinolones.

### 2.3. Sales of Antimicrobials

**Figure 6:**
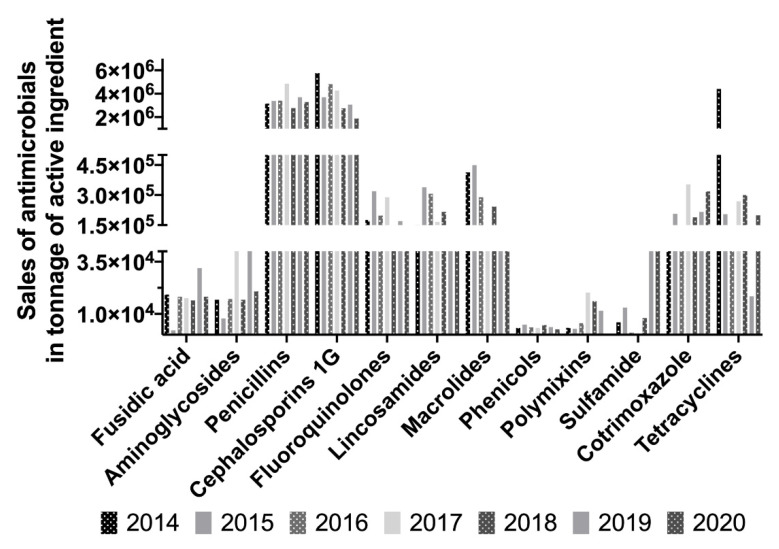
Evolution of antimicrobial sales in tonnage of active ingredient for each antimicrobial class.

Sales of antimicrobials by the pharmacy of the CHUV were monitored during the period of 2014–2020 (Table 5). Over the 6 years of monitoring, the tonnage of antimicrobials sold decreased significantly, to 58.6% in 2020 compared to 2014 (Table 5, Figure 6). This decrease began in 2015. Regarding CIAs, sales of fluoroquinolones began to decline in 2018 after 3 years of increasing sales. Sales of fluoroquinolones decreased to 67.0% in 2020 compared to 2014. The group of antibiotics for which the decreases in sales were the most significant was tetracycline (95.5%), followed by polymyxin (94.4%), macrolides (88.4%), lincosamides (69.6%), and cephalosporins (67.6%). Compared to 2014, the data showed increased sales of aminoglycosides (23.7%), penicillins (4.1%), sulfamides (666.7%), and cotrimoxazole (113.3%).

### 2.4. Animals’ Exposure Levels

**Figure 7:**
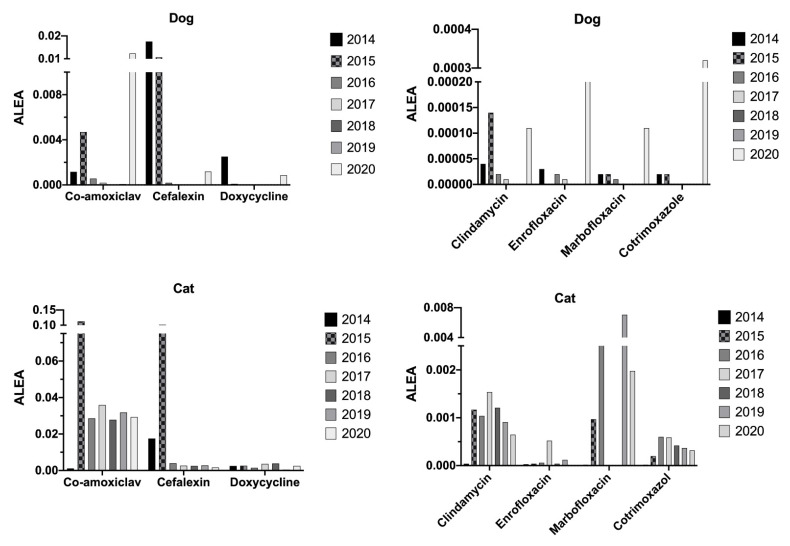
Comparison of the ALEA for dogs and cats by antibiotic molecules from 2014 to 2020.

ALEAs were calculated for oral drugs used for dogs and cats (Figure 7). Overall, the exposure of cats to antibiotics tended to decrease during the analysis period for all antibiotics except marbofloxacin. The exposure of dogs to β-lactamins and doxycycline tended to decrease during the analysis period, which was probably in favor of other molecules, such as clindamycine, enrofloxacin, marbofloxacin, and cotrimoxazole.

## 3. Discussion

The present study involved an analysis of data regarding antimicrobial prescription in a veterinary teaching hospital during a 6-year period. Similar studies have been conducted in other countries. Quantitative analyses of antimicrobial prescriptions were realized in veterinary hospitals in Ontario [35] and Nigeria [36] based on data collected by the hospital pharmacy.

Chirollo et al. (2021) [37] have collected the prescriptions of clinicians at the University Veterinary Teaching Hospital of Naples to analyze the choices of antimicrobials and evaluate the impact of mandates on antimicrobial use. A similar qualitative study was conducted in the veterinary teaching hospital of Naples to describe the use of antimicrobials for companion animals regarding the recommendations on prudent use [38].

The data collected in these studies are less numerous than our study, which may explain why the information extracted from them is qualitatively different. The observations remain close, especially regarding the class of antibiotics most prescribed in companion animal, except for Nigeria [36]. Indeed, in this study, the overall frequency of clinical use of antibiotics on animals increased during the period 2013–2017. Unlike the studies cited above, this study focused on the use of critical antibiotics and the assessment of animal exposure to antimicrobials by calculating the ALEA.

In France, the context of the use of antibiotics in veterinary medicine has changed in the past few years in connection with the implementation of the Ecoantibio Plans and regulatory texts. The aim of the first Ecoantibio Plan was to reduce the risks of antibiotic resistance in veterinary medicine and to safeguard the efficacy of antibiotics. For this purpose, two objectives were established: reduce exposure to antibiotics by 25% in 5 years (measured by the ALEA indicator) and preserve the therapeutic arsenal.

To achieve these objectives, legislative and regulatory action has been taken, including control of the prescription and dispensing of CIAs and publication of a guide for good practice in the use of antibiotics in veterinary medicine. After the success of this first plan, particularly in antibiotics exposure, the Ecoantibio 2 plan was set up, which focused on incentivization by education and communication, promotion of alternatives to antibiotics, and preventive measures for infectious disease.

A report on sales of veterinary medicinal products containing antimicrobials is published every year by the French National Agency for the Health and Safety of Food and the Environment, as well as the National Agency for Veterinary Medicine. This report includes national quantitative data on antibiotic sales and animal exposure to antibiotics by years and species. However, there are no data at the prescription level or on owner dispensation.

Although national monitoring of antibiotic consumption is in place, there is no monitoring of actual antibiotic use at the veterinarian level in France. This is not the case in Switzerland, where prescription data on cats for 2016 and 2018 from 14 private veterinary practices and two university hospitals were collected to evaluate compliance with guidelines [33]. Compliance with Swiss prudent use guidelines was still low, even with the availability of an online tool dedicated to giving specific recommendations on antimicrobial prescription for various diseases and species (antibioticScout.ch, accessed on 8 November 2021) [39]. A second study, conducted later on dogs, demonstrated a significant increase in the proportion of prescriptions in complete agreement with guidelines [40]. This suggests that the provision of a practical, simple, and easily accessible online tool might improve the prudent use of antimicrobials. The use of this tool during the training of veterinarians should help instill good prescription habits.

Veterinary hospitals are structures in which the use of antibiotics is important due to the number of animals that are treated. At the CHUV of the veterinary school of Lyon, the animals received are numerous and are often referred cases. Thus, it is necessary to set an example to ensure that future veterinarians adopt good practice in antibiotic use in connection with the basic education that is carried out. This study provides information on the situation to improve antibiotic prescription and to inform clinicians.

The increase of overall prescriptions during the study period (2014–2020) is due to the systematization of the use of software to write prescriptions (first for companion animals and later for horses). This tool was implemented in 2012–2013 in the CHUV and it is only dedicated to French veterinary schools. The number of caseloads in 2020 is probably correlated with the COVID-19 crisis and containment measures. Those measures have also had repercussions on the possibility of performing antibiograms, as shown in Figure 5. Despite this health crisis, it is interesting to see that the proportion of antibiotics prescriptions decreased in 2015 due to the publication of legislative rules for using antibiotics in veterinary medicine. This phenomenon was also described by Weese JS (2006), who suggest that the implementation of antimicrobial use guidelines was an explanatory factor for the decreasing overall use of antimicrobials [35].

A decrease in the frequency of CIA prescriptions was observed one year later, in 2016, following the publication of regulatory text establishing the list of CIAs and the conditions for their prescription. Efforts to reduce the use of CIAs were maintained until 2019, but a return to a level of prescriptions equivalent to 2014 was observed in 2020. Factors involved in this increase are difficult to find, but it could be associated with the increase of complex referral cases with previous therapeutic failure and antimicrobial resistance development in containment measures. Indeed, these complex cases are frequent in CHUV.

The impacts on patient illness and death were not assessed. Moreover, this study focused only on prescriptions given to owners. Antibiotics administration in the hospital was not include in the analysis. This explains why the oral route is the main route of administration. Parenteral presentations tend to be more prescribed, but there are prescribed for off-label use; for example, fluoroquinolones are administered by auricular route for local otitis treatment to improve antibiotic concentration.

Regarding molecules, β-lactamin was the main group prescribed to companion animals, which is correlated with the availability of these drugs, as well as cotrimoxazole, tetracyclines, and macrolides for many presentations. These results are consistent with those described by Italian universities [37,38], which report that penicillins and cephalosporins are the most prescribed antibiotics in cats and dogs, respectively. Cotrimoxazole prescriptions increased, especially in 2020, due to an increase of use in dogs and horses. The broad spectrum of cotrimoxazole and the recommendation to decrease the use of aminopenicillins might be a hypothesis for this spectacular increase. Moreover, the availability of antibiotics for equines is very limited due to toxicity and the small number of authorized antimicrobials. Prudent use of antimicrobials consists of preserving the use of CIAs (last generation of fluoroquinolones and cephalosporines). Thus, the remaining options in equine medicine are only tetracycline, penicillins, macrolides (with caution due to the toxicity of these three groups), aminoglycosides or colistin (more often used by parenteral route of administration), and cotrimoxazole. It is important to note that the use of antibiotics licensed for human use is very limited, mainly for ophthalmic preparations. Off-label use of human medicinal products is not in agreement with recommendations of prudent use.

French regulations require that CIA prescriptions be justified by the results of sensitivity tests. The absence of these results can only be justified by the impossibility of taking a sample. Prescription in an emergency situation without sensitivity data is tolerated provided that the treatment is re-evaluated after 4 days following analysis of the bacteriological sensitivity results and the clinical context. Thus, all CIA prescriptions are checked to verify the availability of antibiograms.

The results showed a rapid increase of sensibility results from 2015 to 2019. The number of CIA prescriptions that are not associated with sensitivity outcomes decreased over this period. In 2020, data showed fewer antibiogram requests, probably due to the COVID-19 pandemic situation and difficulties in maintaining laboratory and clinical activities. Surprisingly, among the responses to the questionnaire on the rationale for the use of CIAs, the absence of non-CIA adapted antibiotics is very often cited (70.0% in 2019). The interpretation of these data was difficult, and might be done case by case to appreciate this fact, even if it is mentioned in the regulatory text as a possible justification.

However, when the bacteriological results were analyzed, we found that *P. aeruginosa* was isolated very often, mostly in the context of canine ear infections. This bacterium’s many natural resistances to non-critical antibiotics and its location are two arguments in favor of prescribing CIAs by a general route of administration. *Staphylococcus* spp. were also frequently isolated, but the frequency of MRSA was not evaluated.

Regarding the sales of antimicrobials in tonnage of active ingredients, the results are very satisfactory, with a decrease of 58.6% in 2020. The percentage decrease in 2016 of 34.9% was higher than expected by the first National Eco Antibio plan for 2012–2016 (25%) and higher than that achieved by France between 2014 and 2016 for all species (18.16%) [41]. The tonnage of antibiotics sold for dogs and cats in France between 2013 and 2016 was stable. Concerning fluoroquinolones, results were less satisfactory, with an increase of sales in 2016 to 12.3%.

Despite the level of fluoroquinolone prescriptions in 2020 being comparable to 2014, sales have fallen sharply. This is probably linked to regulations that limit the duration of fluoroquinolone prescriptions to 1 month, with the need to repeat a clinical examination to extend the treatment, thus increasing the number of prescriptions. As fluoroquinolones are often prescribed in association with complex dermatological pathologies, the duration of treatment often exceeds one month. This interpretation confirms the need to analyze all available drug-related data (prescription and sales) to understand antibiotic use. However, a more in-depth analysis of prescriptions in terms of dosage and duration of treatment remains complex and challenging.

Sales of antibiotics to animal owners provide an indirect indicator of their final use. Detailed analysis of the data tends to show a change in the distribution of antibiotics sold in relation to prescriptions. Sales of tetracyclines have decreased significantly since 2014, probably in favor of sulfamides due to their very broad spectrum. Sales of cephalosporins have also fallen very rapidly, with a consequent slight increase in the sales of penicillins (especially aminopenicillins). The increase in sales of aminosides is probably related to their primarily local use. Indeed, regulations call for limiting the use of the oral route in favor of local routes.

Finally, animal exposure to antibiotics was evaluated by calculating the ALEA index. This index was established by the French Agency for Veterinary Medicinal Products and is considered the best indicator. In order to obtain a good representation of animal exposure, we collected all data on animal weight and not a standard weight per individual [41]. The results seemed surprising, with an overall increase in ALEA in 2020 (21.1%). However, when considering ALEA by species, the data suggest a decrease in dog exposure to overall antibiotics. This decrease was associated with an increase in feline overall antibiotic exposure. Thus, it was necessary to investigate ALEA by antibiotic groups and species to better understand the trend during the period studied.

As already discussed regarding prescription and antibiotic sales, fluoroquinolones were not a concern, due to a decrease in animal exposure. Again, the increase in complex cases admitted to the hospital may be an explanation for this exception. This study focused on the use of antibiotics in a specific context of a teaching veterinary hospital regarding national data. Globally, the data obtained were very satisfactory and correlated with the consideration of the regulation from its inception.

This is the first time that such a study has been conducted in a hospital while taking into account prescription data, sales data, and the availability of bacteriological results. The interpretation of the results was specific to the type of establishment and cannot be transposed to other activities in medical practice. National reports did not distinguish use regarding the context or analyze it in detail. These data could have been analyzed according to medical sectors, but for reasons of respect for the clinicians, it was preferrable to carry out an overall analysis at the hospital level. Moreover, as in other studies [37,38], it would be appropriate to reconcile these data with clinical data, or at least the anatomical system and recommendations of antimicrobial prudent use. This type of analysis might be conducted over a shorter period of time, given the number of prescriptions, or on cumulated data over several years [37].

The analysis of the data is complex because it is based on a manual analysis using Excel files, as the prescription software tools do not allow this type of study to be carried out. The implementation of simpler tools that are easy for clinicians to use could improve knowledge of antibiotic use. Moreover, this would allow us to better meet the expectations of the authorities.

## 4. Materials and Methods

### 4.1. Study Site

The CHUV is the veterinary teaching hospital of Vetagro Sup, one of the four French veterinary schools. Veterinary schools are under the Ministry of Agriculture, Agri-food, and Forestry. The veterinary teaching hospital is organized into three separate clinical departments according to the target species: one for companion animals and its emergency facility, one for horses and its emergency facility, and a hospital for cattle and small ruminants. In 2019, 92% of the teaching staff were qualified veterinarians, and 42% of them were recognized specialists (EBVS or DESV).

### 4.2. Data Collection of Dispensation and Prescription of Antimicrobials

This study was conducted from 2014 to 2020, and data were obtained via export from our management software. Extracted data were provided in Microsoft Excel format. Pharmacy records at the CHUV were searched for drug-dispensing data and drug prescriptions, which were evaluated in terms of antimicrobial class or the individual antimicrobial when only one particular class was used. Combinations of antimicrobials were also analyzed.

In each prescription, the following data were considered: class of antibiotic prescribed, dosage regimen, route of administration, species, and weight of the animal. If an animal was prescribed the same antibiotic multiples times, the prescription was kept to evaluate animal exposition, but the individual was counted only once. The combinations of amoxicillin with clavulanic acid and of sulfamides with trimethoprim were specifically analyzed under the names “co-amoxiclav” and “cotrimoxazole”, respectively (independently of the sulfamide derivative). All routes of administration were considered, and antibiotic administrations within the hospital were not registered.

### 4.3. Prescription Analysis

All prescriptions entered in the software were exported in Excel format. Each prescription is identified by a unique number and includes species, weight, presentation, and dosage regimen. Once all the prescriptions were exported, a manual analysis was performed to extract the prescriptions containing antibiotics for any route of administration. As CIAs prescription needs to be justified, clinicians should answer a questionnaire before prescribing them; however, it was possible to skip this step.

### 4.4. Calculation

Calculations were based on the methodology proposed by the French Agency for Veterinary Medicinal Products.

- Antibiotics dispensed by the pharmacy

Data on the sale of medicinal products was extracted in Excel format for each year, and then the antimicrobials were extracted manually. The sales data included presentation, number of units dispensed, species, date of dispensation, and the weight and unique identification number of the animal. The antimicrobial group and molecule contained in each presentation were affected. The weight of antimicrobials dispensed was calculated by multiplying quantitative composition of the active ingredient for each presentation by the number of units sold. For active ingredients expressed in international units, a conversion coefficient from OIE recommendations was used. The quantities of antimicrobials sold expressed in milligrams were presented by class of antimicrobials or by species, based on data extracted.

- Animal level of exposure to antimicrobials (ALEA)

ALEAs were calculated according to the method proposed by the French Agency for Veterinary Medicinal Products, based on the recommendations of the European Surveillance of Veterinary Antimicrobial Consumption. Sales data from the pharmacy of CHUV were used. This indicator is calculated by dividing the body weight of treated animals (real data extracted) by the total weight of animals that come to the hospital and are likely to be exposed to antibiotics. The ALEA indicator has no units and is based on the assumption that all the antimicrobials sold during the year are administered to animals in the hospital during this year.

The body weight treated for a given drug was calculated by dividing the weight of antimicrobials dispensed in milligrams of active substance by the dose required to treat one kilogram of typical animal over the entire duration of treatment. It was subtracted from the total mass of the antibiotic dispensed by the drug as follows:

$$\mathrm{Body}\;\mathrm{weight}=\frac{\mathrm{Weight}\;\mathrm{quantity}\;\mathrm{dispensed}}{\mathrm{Maximum}\;\mathrm{dose}\;\mathrm{for}\;\mathrm{the}\;\mathrm{entire}\;\mathrm{treatment}\;\mathrm{period}}$$

For each drug and each species, the dosage selected is the one defined in the marketed authorization for the considered species in mg of antimicrobials per kg of body weight treated. When multiple doses were possible, the highest dose was chosen for the drug’s main indication. When multiple treatment durations were possible, the longest treatment duration was chosen.

$$\mathrm{ALEA}=\frac{\mathrm{Body}\;\mathrm{weight}\;\mathrm{treated}}{\mathrm{Number}\;\mathrm{of}\;\mathrm{animals}\;\mathrm{in}\;\mathrm{the}\;\mathrm{hospital}\;\mathrm{by}\;\mathrm{year}\;\times \;\mathrm{Weight}\;\mathrm{of}\;\mathrm{total}\;\mathrm{animals}}$$

The weight of animals that came to the hospital was extracted for each animal by year. If an animal came several times, it was counted only once, on the basis of the average weight.

### 4.5. Data Organization

Data were analyzed with Excel software for MacOS (version 16.48). Descriptive statistics (frequencies and proportions) were used to summarize the data. The frequencies of prescription across the years were compared using Fisher’s exact test, with Prism 9 software.

## 5. Conclusions

To the author’s knowledge, this is the first time such a study has been conducted in a teaching veterinary hospital in France. This study tend to assess this study tends to show that clinicians take into account the recommendations and regulatory texts in antimicrobial prescriptions. It would be interesting to develop a collaboration between clinical practice and teachers in pharmacy, pharmacology, and bacteriology in veterinary teaching hospitals, which already exists in human hospitals. This could be done by creating an internal antibiotic use committee with an antibiotic manager. This would allow for continued vigilance regarding the proper use of antibiotics and surveillance of antibiotic resistance in this particular context. Indeed, bacterial resistance has not been studied sufficiently, and it would be interesting to explore the evolution of the resistance of bacteria present at the CHUV with regard to the use of antibiotics.

## Figures and Tables

**Table 1:** General data about medical consultation, caseload, and prescription at the teaching hospital of the Veterinary School of Lyon (France). ^a^ Statistically significant frequency variation with 2014 as the reference year (*p* < 0.05).

	2014	2015	2016	2017	2018	2019	2020	Mean	SD
**Number of medical visits ^1^**	20,270	20,300	20,144	18,117	18,827	18,795	14,676	18,732.7	1986
**Number of animals**	8599	8508	8569	8147	8372	7900	6383	8068.29	784.5
**Number of animals for which at least one prescription has been established (all drugs) ^2^**	3255 (37.8%)	5223 (61.4%)	5529 (64.5%)	5497 (67.5%)	5840 (69.7%)	5859 (74.2%)	4441 (69.6%)	5092 (63.5%)	941 (12.1%)
**Number of animals with at least one antibiotic prescription ^2^**	1264 (14.7%) ^a^	1438 (16.9%) ^a^	1374 (16.1%) ^a^	1347 (16.5%) ^a^	1580 (18.9%) ^a^	1814 (22.9%) ^a^	1162 (16.9%) ^a^	1426 (17.6%)	216 (2.7%)
**Total number of prescriptions (all drugs)**	5033	8296	8733	8894	10,725	11,107	8180	8710	1992
**Number of prescriptions with at least one antibiotic ^3^**	1572 (31.2%)	1995 (24.0%) ^a^	1855 (21.2%) ^a^	1944 (21.9%) ^a^	2484 (23.2%) ^a^	2637 (23.7%) ^a^	1883 (23.0%) ^a^	2053 (24.0%)	375 (3.3%)
**Number of prescriptions with a single antibiotic prescribed ^4^**	1390 (88.4%)	1669 (83.7%)	1598 (86.1%)	1656 (85.2%)	2029 (81.7%)	2146 (81.4%)	1516 (80.5%)	1715 (83.9%)	273 (2.9%)
**Number of prescriptions with multiple antibiotics prescribed ^4^**	182 (11.6%)	326 (16.3%) ^a^	257 (13.8%)	288 (14.8%) ^a^	457 (18.4%) ^a^	491 (18.6%) ^a^	367 (19.5%) ^a^	338 (21.8%)	110 (16.4%)
**Number of prescriptions with at least one CIA ^4^**	132 (8.4%)	191 (9.6%)	109 (5.9%) ^a^	152 (7.8%)	191 (7.7%)	160 (6.1%) ^a^	159 (8.4%)	156 (7.6%)	32 (1.53%)
**Number of prescriptions with only one CIA (without other antibiotics) ^5^**	75 (56.8%)	83 (43.5%)	83 (76.1%)	78 (51.3%)	176 (92.1%) ^a^	69 (43.1%)	133 (83.7%) ^a^	98 (63.7%)	38.6 (19.8%)
**Number of prescriptions with one CIA and other antibiotics ^5^**	57 (43.2%)	108 (56.5%)	26 (23.9%) ^a^	74 (48.7%)	15 (7.9%) ^a^	91 (56.9%)	26 (16.3%) ^a^	57 (36.5%)	35 (19.8%)
**Number of prescriptions per animals**	1.5	1.6	1.6	1.6	1.8	1.9	1.8	1.7	0.1
**Number of antimicrobial prescriptions per animals**	1.2	1.4	1.4	1.4	1.6	1.5	1.6	1.4	0.1

^1^ without farm animals; ^2^ percentage expressed in relation to the total of number animals; ^3^ percentage expressed in relation to the total of prescription; ^4^ percentage expressed in relation to the total of prescription containing at least one antibiotic; ^5^ percentage expressed in relation to the total of prescription containing at least one antibiotic.

**Table 2:** Distribution of prescriptions by species regardless of drugs. Values in parentheses refer to the number of antimicrobial prescriptions. Percentages refer to the frequency of antimicrobial prescription in the corresponding species. ^a^ Statistically significant frequency variation with 2014 as the reference year.

	2014	2015	2016	2017	2018	2019	2020	Mean
**Dogs**	2113 (830)	3064 (1020)	3016 (966)	2783 (791)	2855 (810)	2840 (885)	2277 (646)	31.57
	39.3%	33.3% ^a^	32.0% ^a^	28.4% ^a^	28.4% ^a^	31.2% ^a^	28.4% ^a^	
**Cats**	1089 (399)	2065 (378)	2637 (352)	2240 (290)	2236 (352)	2174 (358)	1753 (275)	18.43
	36.6%	18.3% ^a^	13.3% ^a^	12.9% a	15.7% ^a^	16.5% ^a^	15.7% ^a^	
**Horses**	ND	7 (0)	60 (24)	403 (242)	653 (390)	746 (543)	333 (208)	59.00
	ND	0.0%	40.0% ^a^	60.0% ^a^	59.7% ^a^	72.8% ^a^	62.5% ^a^	
**Uncommon pets**	41 (29)	84 (13)	85 (31)	68 (23)	86 (22)	90 (25)	75 (28)	32.29
	70.7%	15.5% ^a^	15.3% ^a^	33.8% ^a^	25.6% ^a^	27.8% ^a^	37.3% ^a^	

**Table 3:** Responses to the automatic questionnaire generated by the software for each prescription of CIAs.

	2016	2017	2018	2019	2020
Number of completed questionnaires	25	94	168	145	96
	22.9%	61.8%	57.1%	90.6%	60.4%
Number of “susceptibility test pending results” responses	10	33	47	41	38
	9.2%	21.7%	24.6%	25.6%	23.9%
Number of “sampling not feasible” responses	2	13	36	26	45
	1.8%	8.6%	18.8%	16.3%	28.3%
Number “susceptibility test results less than 3 months” responses	2	58	104	104	74
	1.8%	38.2%	54.5%	65.0%	46.5%
Number “probabilistic use of antibiotic” responses	6	22	59	59	61
	5.5%	14.5%	30.9%	36.9%	38.4%
Number “no adapted antibiotic available” responses	19	81	128	112	96
	17.4%	53.3%	67.0%	70.0%	60.4%

**Table 4:** Distribution of the most frequently isolated bacteria associated with an CIA prescription.

		2016	2017	2018	2019	2020
**Gram −**	***Pseudomonas aeruginosa***	9	17	34	32	19
	***Pseudomonas fluorescens***	2	2	2		1
	***Escherichia coli***	2	2	15	8	3
	***Morganella morganii***	1	1			
	***Klebsielle pneumoniae***	1			7	1
	***Proteus mirabilis***	3	5	4	5	1
	***Chryseobacterium indologenes***			2		
	***Pasteurella pneumotropica***			1		
	***Serratia marcescens***	1		2		
	***Acinetobacter baumanii***		2	2		
**Gram +**	***Staphylococcus* spp.** ***(coagulase positive)***	5	18	23	19	5
	***Staphylococcus* spp.** ***(coagulase negative)***	2		5		
	***Streptococcus equii*** **ssp.** ***equii***				2	1
	***Streptococcus equii*** **ssp.** ***zooepidemicus***			9	2	1
	***Streptococcus dysglactiae***					1
	***Streptococcus canis***	1				
	***Enterococcus cloacae***	1	1	1	5	
	***Enterococcus faecalis***	1	1			
	***Clostridium perfringens***				1	
	***Rhodococcus equi***				3	
	***Aerococcus viridans***			1		

**Table 5:** Percentage change in antimicrobial sales in tonnage of active ingredient for each antimicrobial class or molecules compared to 2014.

	2015	2016	2017	2018	2019	2020
**Fusidic acid**	↓89.3%	↓5.2%	↓8.9%	↓14.5%	↑65.7%	↓5.5%
**Aminoglycosides**	↓53.7%	↑2.2%	↑402.6%	↓0.1%	↑189.1%	↑23.7%
**Penicillins**	↑6.8%	↑7.7%	↑53.5%	↓12.5%	↑17.1%	↑4.1%
**Cephalosporins 1G**	↓35.7%	↓16.5%	↓25.9%	↓52.1%	↓46.8%	↓67.6%
**Fluoroquinolones**	↑81.8%	↑12.3%	↑64.5%	↓22.3%	↓3.2%	↓67.0%
**Lincosamides**	↑125.3%	↑103.5%	↑10.1%	↑43.4%	↓56.4%	↓69.6%
**Macrolides**	↓8.6%	↓30%	↓67.6%	↓41.3%	↓64.8%	↓88.4%
**Phenicols**	↑47.7%	↑9.2%	=0%	↑42.5%	↑13.8%	↓20%
**Polymixins**	↓13.7%	↑68.3%	↑512.1%	↑387.3%	↑247.7%	↓94.4%
**Sulfamide**	↑116.7%	↓83.3%	↓100%	↑33.3%	↑600%	↑666.7%
**Cotrimoxazole**	↑38.0%	↓24.1%	↑136.9%	↑26.7%	↑44.7%	↑113.3%
**Tetracyclines**	↓95.3%	↓97.2%	↓93.9%	↓93.2%	↓99.6%	↓95.5%
**Total of antibiotic per year (mg) and% of variation since 2014**	↓39.5%	↓34.9%	↓26.7%	↓53.2%	↓47.3%	↓58.6%
